# A Review of Neurostimulation for Epilepsy in Pediatrics

**DOI:** 10.3390/brainsci9100283

**Published:** 2019-10-18

**Authors:** Keith Starnes, Kai Miller, Lily Wong-Kisiel, Brian Nils Lundstrom

**Affiliations:** 1 Department of Neurology, Mayo Clinic, Rochester, MN 55905, USA; starnes.donnie@mayo.edu (K.S.); wongkisiel.lily@mayo.edu (L.W.-K.); 2 Department of Neurologic Surgery, Mayo Clinic, Rochester, MN 55905, USA; miller.kai@mayo.edu

**Keywords:** pediatric neurostimulation, neuromodulation, drug-resistant epilepsy, vagus nerve stimulation, responsive neurostimulation, deep brain stimulation, chronic subthreshold cortical stimulation, transcranial magnetic stimulation, transcranial direct current stimulation

## Abstract

Neurostimulation for epilepsy refers to the application of electricity to affect the central nervous system, with the goal of reducing seizure frequency and severity. We review the available evidence for the use of neurostimulation to treat pediatric epilepsy, including vagus nerve stimulation (VNS), responsive neurostimulation (RNS), deep brain stimulation (DBS), chronic subthreshold cortical stimulation (CSCS), transcranial magnetic stimulation (TMS) and transcranial direct current stimulation (tDCS). We consider possible mechanisms of action and safety concerns, and we propose a methodology for selecting between available options. In general, we find neurostimulation is safe and effective, although any high quality evidence applying neurostimulation to pediatrics is lacking. Further research is needed to understand neuromodulatory systems, and to identify biomarkers of response in order to establish optimal stimulation paradigms.

## 1. Introduction

### 1.1. Neurostimulation for Epilepsy in Pediatrics

Anti-seizure medications (ASMs) are considered the first-line therapy for epilepsy. Patients who do not become seizure-free after using two appropriately-chosen ASMs at therapeutic doses are unlikely to be seizure-free by using medications alone, and the epilepsy is termed pharma-co-resistant [1,2,3]. Other treatment options include diet therapy, surgical resection, and neurostimulation.

Neurostimulation refers to the application of electricity to affect the central nervous system with the goal of reducing seizure frequency and severity. Though this idea has existed for more than a century [4], neurostimulation has become increasingly widespread since vagus nerve stimulation (VNS) gained United States Food and Drug Administration (FDA) approval in 1997 [5]. Various modalities exist, ranging from non-invasive to invasive. Some target the seizure-onset zone (SOZ) for the individual patient, while others target more general regions thought to affect seizure-related neural networks. Some devices provide continuous stimulation (open-loop), whereas others sense brain activity, and deliver stimulation based upon detected events (closed-loop) [6,7,8].

Despite the growing number of devices and neurostimulation-related literature, relatively little is known about the underlying mechanisms and parameter optimization. The effect of stimulation on seizure burden is typically not readily apparent, and biomarkers to assess the effect of neurostimulation have not been well-delineated.

Optimal parameter choices for neurostimulation are rarely if ever known. In general, randomized-controlled trials of neurostimulation devices in pediatric populations are lacking.

In this review, we discuss the available devices for neurostimulation, and the evidence to support their use for epilepsy in pediatric patients. We consider possible mechanisms of action, as well as safety concerns. Finally, we propose a framework for selecting among the available options, depending upon patient characteristics and preferences.

### 1.2. Invasive vs Non-Invasive

**Figure 1:**
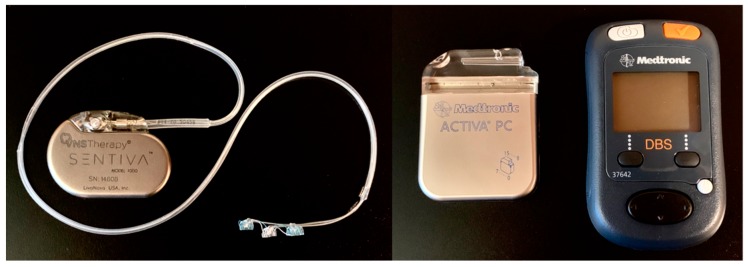
Examples of vagus nerve stimulation (VNS) and deep brain stimulation (DBS) devices. VNS internal pulse generator or neurostimulator (left side of left panel) with extension and coil electrodes (right side of left panel). DBS neurostimulator (left side of right panel) and patient programmer (right side of right panel).

We use “invasive” to refer to devices that require a surgical procedure to implant. Namely, these include VNS, deep brain stimulation (DBS), responsive neurostimulation (RNS) and chronic subthreshold cortical stimulation (CSCS). In general, stimulation devices include an internal pulse generator (IPG), or neurostimulator, that supplies electricity via an extension to implanted electrodes (Figure 1). The generator for VNS, DBS and CSCS is implanted into the subclavicular region of the thoracic chest wall, while the generator for RNS is implanted intracranially.

“Non-invasive” refers to neurostimulation devices that do not require the permanent implantation of a device. In other words, stimulation is applied externally and intermittently to the nervous system. For the purposes of this review, we will consider transcranial magnetic stimulation (TMS) and transcranial direct current stimulation (tDCS).

### 1.3. Closed-Loop vs. Open-Loop

Most neurostimulation devices that exist today are open-loop; that is, they deliver continuous stimulation in a pre-defined pattern without feedback. In contrast, RNS is closed-loop, meaning that stimulation is delivered only when certain conditions are detected by the device [9]. More recent models of VNS have a sensing component, by which stimulation is provided when tachycardia is present [10].

**Figure 2:**
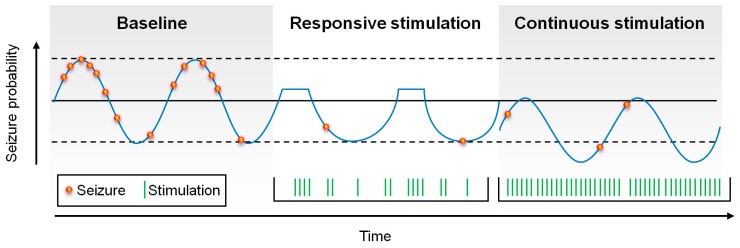
Schematic of the possible affects upon seizure probability by responsive and continuous stimulation. Responsive stimulation may abort seizures in real time, reducing the time in which the patient is at high-risk for seizures, whereas continuous stimulation may “shift” seizure probability down by modulating broader epileptogenic networks.

Seizure probability oscillates, based upon circadian and patient-specific cycles [11,12]. Conceptually, closed-loop stimulation has been thought to work by aborting seizures in real-time, or at least by responding to seizure-related electrical activity, reducing the time that patients spend in highly seizure-prone states. In contrast, open-loop stimulation may affect, not just seizure-prone states, but all states, thus reducing global seizure probability (Figure 2).

### 1.4. Review Methodology

Studies were identified by PubMed searches using the terms “neurostimulation”, “vagus nerve stimulation”, “responsive neurostimulation”, “deep brain stimulation”, “chronic subthreshold cortical stimulation”, “transcranial magnetic stimulation” and “transcranial direct current stimulation”, in combination with the term “pediatrics.” The studies were then reviewed for relevance to the use of neurostimulation in pediatric patients. There has been only a single randomized controlled trial for neurostimulation in pediatric epilepsy [13]. The remainder of the evidence is limited to case reports and case-control and cohort studies. As a result, studies including adult patients are discussed where pediatric data is lacking.

## 2. Vagus Nerve Stimulation

### 2.1. Introduction

Vagus nerve stimulation (VNS) gained US FDA approval for intractable focal epilepsy in 1997, and is now approved for patients aged four years and older. It is the most well-studied neurostimulation modality in pediatrics. A cuff is threaded around the vagus nerve and connected to a VNS generator that is typically implanted superficial to the pectoral muscle. Usually the left vagus nerve is chosen to avoid any stimulation of the sinoatrial node, which receives input from the right vagus nerve [14]. Clinical studies suggest that cardiac effects with right-sided vagus nerve activation are typically minor [15]. In addition to pre-defined ongoing stimulation sequences, VNS affords the ability for patients or their families to activate stimulation by swiping a magnet over the device. Significantly more than 100,000 VNS devices have been implanted worldwide for the treatment of epilepsy [16].

### 2.2. Mechanism

VNS is theorized to modulate hyper-excitable brain regions by increasing activity in the nucleus tractus solitarius (NTS) and its downstream projections to the limbic system and thalamus [16]. The NTS then projects to the locus coeruleus and to the raphe nuclei, and VNS increases the production of norepinephrine (NE, aka noradrenaline (NA)) and serotonin, which have been shown to have antiepileptic effects [17].

### 2.3. Evidence

Penry, et al. reported the first human implant of VNS in 1990 [18]. A prospective, randomized, controlled pilot study was performed enrolling 114 patients (EO3), showing a 39% responder rate after three months in patients receiving “high-intensity” stimulation (typical parameters: 1.5 mA output current, 30 Hz frequency, 500 µs pulse width, 30 s/5 min on/off time) and 19% in patients receiving “low-intensity” stimulation (1.25 mA, 1 Hz, 130 µs, 30 s/90 min on/off time) [19]. A subsequent multicenter trial of 199 patients (EO5) showed similar response rates [20]. Interestingly, another RCT comprised of 41 pediatric patients comparing high-output stimulation (maximal parameters: Output current 1.75 mA, pulse width 500 µs, 30 Hz frequency, 30 s/5 min on/off time) and low-output stimulation (0.25 mA, 100 µs, 1 Hz, 14 s/60 min on/off time) showed a greater than 50% reduction in seizure frequency in approximately 20% of patients, with no significant difference reported between groups [13].

A 2015 Cochrane review included these studies, as well as an earlier pilot study and another multi-center trial comparing three treatment arms (rapid, medium and slow duty cycling) [21,22], in total comprising 439 participants. The review concluded that there was a statistically significant difference in response between high and low stimulation groups [23]. A meta-analysis of 74 studies with 3321 patients reported that seizures were reduced by 50% or more in approximately 50% of patients at last follow-up [24].

Other studies have shown similar levels of efficacy in pediatric and adult patients in VNS [25]. The rate of response improves over time. 440 patients from five of the early clinical trials were followed for three years with 44% responders [26]. A large retrospective European study that examined 24-month outcomes in 347 children implanted with VNS demonstrated a 44% responder rate [27]. Another retrospective review of 436 patients implanted at a single center showed a 64% responder rate at a mean 5-year follow-up [28].

VNS may also be effective in severe forms of drug-resistant epilepsy. Published 2013 guidelines examined 14 studies of VNS in Lennox-Gastaut syndrome encompassing 481 children. 55% were responders, although those authors note significant variability in results across centers [29].

There have been several generations of VNS devices, with the more recent versions including more advanced features, such as the ability to deliver higher-intensity stimulation in response to elevations in heart rate, based upon the observation that 82% of epilepsy patients experience ictal tachycardia [30]. One study suggested improved efficacy of this device in patients undergoing battery replacements, as well as in new implants [10]. Other quality-of-life improvements of new models include the ability to pre-program stimulator settings to increase automatically, limiting the number of required follow-up appointments after implantation [31].

### 2.4. Safety and Tolerability

VNS is generally well-tolerated. Common non-implantation-related adverse effects include voice changes, dysphagia, coughing, and neck pain [32]. Side effects tend to be related primarily to output current, and to a lesser extent, duty cycling. These parameters can generally be adjusted to patient tolerability [33,34,35]. Evidence supports a relationship between VNS and central and obstructive sleep apnea [36,37,38], and caution should be exercised when using VNS in patients with these conditions. VNS devices in general are compatible with MRI with certain protocols [39].

### 2.5. Discussion

VNS is the best-studied and most widely used of the neuromodulatory modalities for epilepsy in pediatric populations. It is safe, effective and FDA-approved. Data support its use across a variety of epilepsy types.

## 3. Responsive Neurostimulation

### 3.1. Introduction

A responsive neurostimulation (RNS) device is a closed-loop device that delivers targeted stimulation to the putative SOZ. Unlike the other methods of neurostimulation discussed here, RNS was designed to abort seizure activity. It relies on a pre-defined seizure-detection algorithm that triggers stimulation intended to abort seizures or seizure-related activity [40]. The pulse generator is implanted into the cranium. Detection and stimulation parameters are clinician-adjustable, based upon individual patient characteristics [9].

RNS (NeuroPace, Inc., 455 N Bernardo Ave, Mountain View, CA 94043, United States) was FDA-approved in 2013 for the treatment of focal drug-resistant epilepsy in adults [40]. Bergey, et al. reported a blinded, randomized, controlled trial, showing a 53% median seizure reduction two years after implantation in 256 adult patients [41]. Responder rates ranged from 50%–61% over six years, and median percent reduction in seizures was 66% by year six. As with other stimulation approaches, responder rates improved over time [42].

### 3.2. Mechanism

The design of RNS was based upon the observation that delivering stimulation can abort after-discharges elicited during electrical stimulation for functional mapping in patients undergoing intracranial monitoring [43]. Although initially conceived as a way to abort seizure activity, responsive stimulation may primarily work by altering the plasticity of relevant neural networks [44]. For example, RNS may function by suppressing cortical synchronization, even in regions distal from the area of stimulation [45]. There is evidence that the benefits of RNS are due to indirect prevention, rather than triggered seizure inhibition. [46]

### 3.3. Evidence

RNS use is not presently FDA-approved in children, and published experience is limited. Singhal et al. reported the successful treatment of a 16-year-old girl with a seizure onset in the eloquent cortex of the left temporal neocortex, with a reduction from daily seizures to auras only a few times per week at the six-month follow-up [47]. Kokoszka et al. reported using RNS in a 14-year-old with independently hemispheric seizure onset and a 9-year-old with seizure onset in the eloquent cortex in the left frontal and parietal lobes, with greater than 80% reduction in seizure frequency in both patients [48].

### 3.4. Safety and Tolerability

RNS is well-tolerated. Risk of infection has been reported as 3.7% per procedure [42]. Intracranial hemorrhage was reported in 4.7%, and lead damage in 2.6% of procedures [9,41]. Other complications that are not implantation-related are uncommon, and no significant difference in adverse events was seen across sham or treated groups [49]. Serious adverse event rates are similar to those of other intracranial devices or epilepsy surgery [50,51]. The RNS system is not presently MRI compatible [52]. Studies have shown no significant impairment of mood or cognition, with some patients experiencing improvements [53,54].

### 3.5. Discussion

At present, RNS is the only commercially-available means of long-term electrocorticography. Clinically, this is useful for tracking seizure detections over time, and for example, determining the laterality of seizure onset when there is bilateral mesial temporal involvement. It thus affords diagnostic utility, and RNS has led to successful resection of a solitary seizure focus in patients who were presumed to have multifocal epilepsy [55,56]. There is limited data to support RNS use in children.

In contrast to DBS, which was used for years for treating movement disorders prior to epilepsy, RNS involves novel hardware placement as well as a new stimulation paradigm. Currently, RNS use in pediatrics is limited to off-label implantations at epilepsy centers familiar with its use in adult patients, and only after achieving expert consensus.

## 4. Deep Brain Stimulation

### 4.1. Introduction

Deep brain stimulation (DBS) was FDA-approved for use in intractable epilepsy in patients 18 years of age or older in 2018, following the results of the Stimulation of the Anterior Nucleus of the Thalamus for Epilepsy (SANTE) trial [57], after which adult patients were prospectively followed for five years with a median percent seizure reduction of 69% [58].

For stimulation of the anterior nucleus of the thalamus (ANT) DBS, a permanent generator is implanted superficial to the pectoral muscle, and electrodes are placed into the bilateral ANT. Other targets have included the centromedian nucleus of the thalamus (CMT), the subthalmic nucleus (STN), the globus pallidus, the cerebellum, the hippocampus, the caudate nucleus and the seizure onset zone itself [59]. The CMT has been targeted for the treatment of generalized epilepsy [8,60,61,62,63,64,65]. A recent review by Li and Cook [59] summarized three small (largest sample size *N* = 9) randomized-controlled trials of stimulation of the hippocampus for mesial temporal lobe epilepsy, with all three showing a reduction in seizure frequency (15%–40%) versus placebo [66,67,68]. All patients in the larger study had a >50% reduction in seizure frequency during un-blinded follow-up [69].

### 4.2. Mechanism

The mechanism of DBS is not well understood, but is thought to disrupt networks involved in seizure propagation [70]. The ANT is an important node in the limbic circuit of Papez [71,72]. The exact means through which this effect is mediated is not known—DBS does not functionally “lesion” the target area, and the relative neuromodulatory or neuroinhibitory effects depend upon the parameters of stimulation [62,63]. Stimulation of the ANT and of the CMT can produce generalized responses on scalp EEG and reduce interictal discharges [73,74], indicating the influence of thalamic stimulation upon diffuse network activity.

### 4.3. Evidence

There have been no randomized controlled trials for the use of DBS in pediatric patients with epilepsy. A recent systematic review identified case reports totaling 40 patients ages 4–18 who were treated with DBS for a variety of indications [60]. Five (12%) became seizure-free. 17 of 18 (94%) patients with CMT stimulation and 5 of 8 (63%) with ANT stimulation had a greater than 50% reduction in seizure frequency. The remaining 14 patients had stimulation of the subthalamic nucleus, hippocampi, caudal zone incerta, mammillothalamic tract, or posteromedial hypothalamus.

Benabid et al. [75] reported the case of a 5-year-old girl with focal centroparietal cortical dysplasia, who underwent implantation with a permanent electrode in the left subthalamic nucleus, and subsequently had an 80% reduction in the number and severity of seizures. Valentin et al. [76] described two children with generalized epilepsy who underwent stimulation of the bilateral CMT, and one with right frontotemporal epilepsy who underwent stimulation of the ANT for a period of 20–161 h while being monitored with intracranial EEG. Two had a greater than 60% reduction in seizure frequency; the other patient did not improve [76].

Thirteen children with Lennox-Gastaut syndrome were treated with CMT stimulation, with either bilateral or unilateral leads, with the latter occurring when these leads were inaccurately placed [64]. At the 18-month follow-up, all were responders and two were seizure-free.

### 4.4. Safety

In the SANTE trial, the most frequent adverse effect was implant site pain or paresthesias in 23%. Implant site infection was reported in 12.7%, and lead misplacement in 8.2% [58]. A systematic review of DBS identified two adverse events occurring at a significantly higher rate in the active group, compared to sham stimulation: Depression (14.8% vs. 1.8%) and memory impairment (13.0% vs. 1.8%) [77]. However, at the five year follow-up in the SANTE trial, no objective neurobehavioral deterioration was observed [78]. Evidence suggests that anterior nucleus stimulation can be associated with small improvements in cognition and memory [79,80,81].

### 4.5. Discussion

Due to limitations of available data regarding the use of DBS in children, it is unclear to what extent available data in adults can be extrapolated to the pediatric population. More data is available for the use of DBS in pediatric movement disorders, and the device is FDA-approved for dystonia in children [82]. Stimulation parameters in epilepsy and movement can be similar [83]. Safety data are favorable, and may reasonably be extrapolated to epilepsy [84]. Among the 40 patients included in a review of pediatric DBS [60], there were four cases of infection, two of battery skin erosion and one of lead breakage.

However, technical challenges exist. The most common serious adverse effects in adults are infection and lead misplacement [58]. These issues could be more common in pediatric populations, as the head and body grow. Furthermore, safe and effective stimulation parameters have not been established for children. DBS does not preclude obtaining MR imaging, but is protocol dependent [85].

To summarize, DBS is safe, effective and FDA-approved to treat intractable epilepsy in adults, but data is lacking in pediatrics. Patient selection parameters have not been defined. Longitudinal data is limited. Several case reports indicate promise for its tolerability and efficacy in pediatric patients, but no randomized controlled trials to support its use are available in this population.

## 5. Chronic Subthreshold Cortical Stimulation

### 5.1. Introduction

Like RNS, chronic subthreshold cortical stimulation (CSCS) targets the location of seizure onset [86]. However, it is open-loop, similar to VNS and DBS. Continuous stimulation is subthreshold, i.e., delivered in a fashion that preserves existing cortical function [87]. Potential candidates are identified while undergoing intracranial EEG monitoring. If seizure onset is multifocal or involves eloquent cortex, current practice includes 1–3 days of trial stimulation using the existing implanted hardware. Seizure frequency and the frequency of interictal epileptiform discharge are used to assess the potential efficacy of multiple stimulation paradigms. If stimulation appears to be efficacious, permanent leads can be implanted [87].

### 5.2. Mechanism

The precise mechanism by which continuous stimulation lowers seizure probability is unknown. CSCS is based upon prior evidence, suggesting continuous cortical stimulation is safe, and can lower seizure frequency. Velasco, et al. described 10 patients with intractable temporal lobe epilepsy who subsequently went on to have temporal lobectomies, and were implanted with depth and subdural electrodes to identify the extent of the seizure onset zone [88]. The diseased hippocampi were chronically stimulated for 2–3 weeks. The frequency of seizures and interictal discharges showed a progressive decline during continuous stimulation. Pathology showed no evidence of stimulation-related damage [88]. The same group later reported a greater than 90% seizure reduction in two patients with a stimulation of the eloquent motor cortex for about one year [89].

Another report of a patient with seizure onset in the primary motor cortex described continuous stimulation that was well-tolerated for five years, and led to a reduction in seizure frequency of more than 90%, with an elimination of secondary generalization [90].

### 5.3. Evidence

For seven pediatric patients ages 6–17, three (43%) became seizure free, and all had a greater than a 50% reduction in seizure frequency [86]. A subsequent review approximately two years later including these seven pediatric patients (median follow-up 2.8 years) showed that five of this seven were free of disabling seizures in the most recent 3-month period, and that mean seizure frequency reduction was 85% [91].

These high rates are likely to be at least partially explained by selection bias, as CSCS patients typically undergo some trial stimulation prior to permanent implantation [86,92,93]. Valentin et al. also described five children who underwent cortical stimulation for 1–6 days, with four showing greater than 50% reduction in seizure frequency during the time of stimulation [76]. The same group reported a seven-year old boy with frequent multifocal seizures who experienced long-term seizure freedom after four days of continuous temporo-parietal stimulation [94].

### 5.4. Safety and Tolerability

CSCS is well-tolerated [93]. It likely has a rate of device-related complications similar to other implantable devices. Ten of 13 patients reported improvement in life satisfaction [86]. In some cases, neurologic function appears to improve with CSCS [95]. Parameters can be adjusted based on individual patient characteristics. CSCS has been implemented via off-label usage of FDA-approved hardware manufactured by Medtronic (Dublin, Eire and Minneapolis, MN, USA) that is approved for conditional MR imaging.

### 5.5. Discussion

At present, clinical outcome data are retrospective and limited, especially in pediatric populations. Only a few centers have experience with CSCS. Early tolerability data are promising, and it may be reasonable to expect a similar safety profile to other forms of intracranial stimulation, though the surety of this eventuality remains unknown.

## 6. Transcranial Magnetic Stimulation

### 6.1. Introduction and Mechanism

Whereas electrical stimulation is delivered internally in VNS, DBS, RNS and CSCS, in transcranial magnetic stimulation (TMS), specified cortical circuits are modulated by external fluctuations in a magnetic field. This magnetic flux generates intracranial currents, which can excite action potentials and alter cortical excitability. TMS has been utilized for numerous indications, and is FDA-approved for the treatment of major depression, migraine and pre-surgical mapping of motor and language function [96].

### 6.2. Evidence

A 2016 Cochrane review of available evidence for the use of TMS to reduce seizure frequency judged the quality to be low [97]. Fregni et al. performed a randomized clinical trial of active or sham TMS in 21 patients with malformations of cortical development and refractory epilepsy [98]. Patient ages were not individually reported (mean 21.9 ± 8.1 years), but the study included pediatric patients. A reduction in seizure frequency by 72% was seen in the active group, which was significantly more than the sham group in a follow up period of eight weeks [98]. Evidence suggests TMS can reduce the frequency of interictal discharges [99,100].

### 6.3. Safety

There is evidence that TMS is safe [101]. Some patients report mild headaches following treatments. The most significant risk is for generating seizures with TMS, with one review suggesting a 0.14% risk per session [102]. Other adverse effects are rare [101,102].

### 6.4. Discussion

Although TMS is safe, available data have not established the efficacy of TMS for the treatment of pediatric or adult epilepsy. One challenge is that even if efficacious, repeated TMS treatments would likely be required to maintain seizure reduction.

## 7. Transcranial Direct Current Stimulation

### 7.1. Introduction and Mechanism

Transcranial direct current stimulation (tDCS) typically uses two large scalp electrodes (anode and cathode) to deliver constant current to the brain. In general, cathodal stimulation is thought to decrease cortical excitability by stabilizing neuronal membranes, and is commonly employed to target regions of seizure onset [103,104,105,106]. This tDCS has been studied in pediatric patients for attention deficit hyperactivity disorder (ADHD), depression, cerebral palsy and autism spectrum disorder (ASD, among which is Asperger’s Syndrome) [96,107]. A European review determined that tDCS is probably efficacious in fibromyalgia, depression and addiction/craving [108].

### 7.2. Evidence

In epilepsy, an early case series showed a reduction in seizures in 18 children with brain lesion-associated focal seizures [109]. Other case reports describe a significant reduction in interictal discharges outlasting the time of stimulation in numerous children [110,111,112]. One study of 36 children showed a small but statistically-significant reduction in seizure frequency [111]. The same group reported a significant reduction in inter-ictal epileptiform discharges (IEDs) and seizure frequency in 22 children with Lennox-Gaustaut syndrome after five days of cathodal tDCS applied to the primary motor cortex [113]. A double-blinded and sham-controlled crossover study in five pediatric patients with continuous spikes and waves in slow wave sleep (CSWS) did not show a decrease of epileptiform activity with tDCS [114].

The montage used in tDCS defines the direction and field of current flow. Methods of stimulation vary across studies, and multiple electrodes can be used to target the region of seizure onset more precisely [115]. It remains unclear whether targeted stimulation is more efficacious.

### 7.3. Safety

The procedure tDCS is very safe [103], with only mild skin irritation at the site of electrodes being a common side effect. No serious adverse events have been reported in more than 33,000 sessions in human subjects [116].

### 7.4. Discussion

Currently, the level of evidence to support the use of tDCS in epilepsy is limited. Initial studies have shown promising results in limited populations and supported the safety of tDCS. Larger studies are needed to determine efficacy of tDCS for epilepsy, to guide patient selection and also to define stimulation parameters.

## 8. Other Forms of Neurostimulation for Pediatric Epilepsy

Transcutaneous VNS (tVNS) and trigeminal nerve stimulation (TNS) have been investigated in small studies. A trial comparing tVNS at 1 Hz versus 25 Hz showed a reduction from the baseline in the 25 Hz group, but no difference between groups [117].

An initial TNS trial showed a mean seizure reduction in 7 of 13 patients who completed a 12-month trial [118], but a larger randomized controlled trial including 50 patients failed to show significant differences between active and sham groups [119]. In the 35 patients who continued the trial for a year, 31% were responders [120].

## 9. Discussion

There are no established guidelines for the selection of neurostimulation modality in pediatric patients with pharma-co-resistant epilepsy. This mirrors the selection of initial ASM, for which guidelines are also not established. Rather the decision is made upon individual patient characteristics and practical considerations. There are insufficient data at present to recommend the use of TMS or tDCS, and so the following discussion will not include those options. It should be noted, however, that these methods are generally safe, and could be considered in select situations.

In general, neurostimulation is palliative, and rates of prolonged seizure freedom are low. In pediatric populations, high-quality, randomized, controlled trial data is only available in VNS, with an approximate 40% of patients showing a > 50% reduction in seizure frequency at one year [21], though the proportion has been reported as higher in retrospective studies [24,26,27,28]. In adult populations, RNS and DBS have similar 50%–65% responder rates over time [41,59].

VNS is the only stimulation modality that is FDA-approved for the treatment of epilepsy in pediatric patients. It is therefore the first option tried in many cases. There does not appear to be any correlation between the efficacy of VNS and the later efficacy of RNS [41] or DBS [58] for treatment of epilepsy. RNS and DBS, along with VNS, are FDA-approved for use in adults (Table 1).

Data supporting the use of RNS or CSCS for generalized epilepsy is limited or non-existent. Each modality has been effective in focal epilepsy. VNS and DBS are “non-targeted”, and do not require invasive intracranial monitoring to precisely localize seizure onset, whereas RNS and CSCS may require precise identification of seizure onset.

Each device is generally safe. VNS is most likely to be associated with mild side effects. DBS and RNS side effects are usually device-related and relatively rare. Memory and cognition either appear to be not affected or to be potentially improved, particularly with intracranial stimulation of the lateral temporal structures and fornix [122]. With DBS ANT, there has been an increased frequency of subjective memory or mood complaints [57]. CSCS in rare cases may improve neurological function [95]. VNS, DBS ANT and CSCS are MRI-conditional. RNS is not presently MRI-compatible.

Patient age may be another important factor. VNS is approved in ages four and older. Given the potential for leads to move with head growth, this may be a reasonable minimum age for invasive devices.

**Figure 3:**
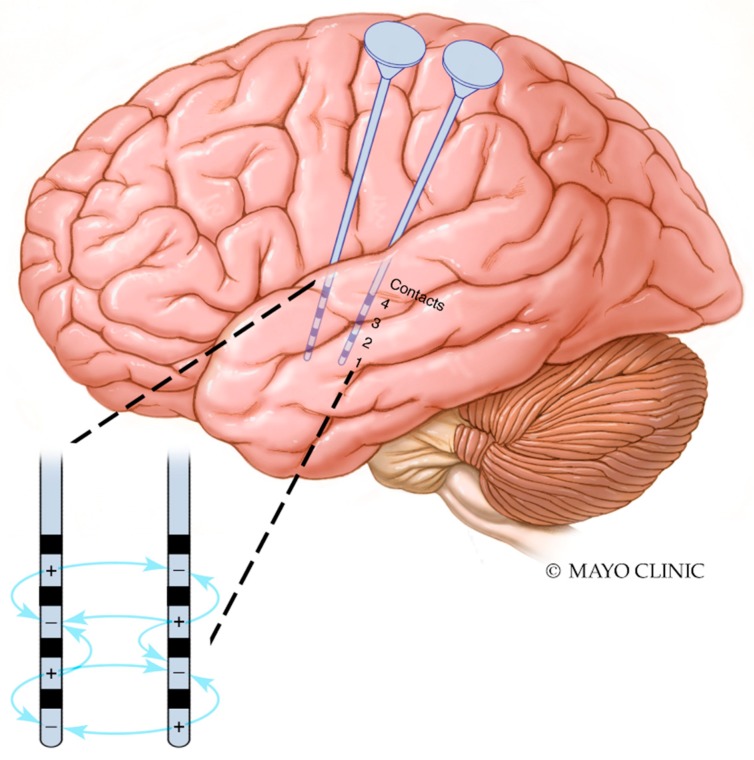
Interaction between cathodal and anodal contacts in proximal electrodes. Interactions between contacts in two temporal depth electrodes are illustrated. Dipoles are generated between contacts on the same electrode as well as between cathodal and anodal contacts on nearby electrodes. For the sake of simplicity, not all possible interactions are shown; dipoles are generated between all cathodes and anodes.

Finally, the parameter space is vast in each device, and there are not yet methods of establishing optimal parameters. A trial-and-error approach is generally chosen, with parameters adjusted when seizures reoccur [123]. In the case of CSCS and other invasive brain stimulation, there must be special consideration of the location of cathodal and anodal contacts. Electrical dipoles are generated not only between stimulating contacts on the same electrode, but also to nearby contacts on other electrodes (Figure 3). It is unknown whether patients who have failed neurostimulation have done due to a failure of the approach, or due to a failure to determine optimal stimulation parameters.

## 10. Conclusions

Neurostimulation for the treatment of pharmacoresistant pediatric epilepsy is safe and effective. Multiple options exist and can be chosen based on individual patient characteristics. Patient selection criteria have not been well-defined. Aside from VNS, which is the only FDA-approved neurostimulation device for children with epilepsy, randomized, controlled trials are lacking for the pediatric population. Larger trials are needed to investigate the efficacy and safety of RNS, DBS, CSCS, TMS and tDCS, as well as other modalities in pediatric populations. There are no guidelines for choosing the best method of stimulation for each patient, and this choice often relies upon provider familiarity.

A method of establishing individualized optimal stimulation parameters has not been established. In the future, a means of estimating seizure probability in real-time may provide the ability to choose the stimulation settings more rationally. This would be aided by an improved understanding of the mechanisms of action and identification of the biomarkers of neuromodulatory responses.

## Figures and Tables

**Table 1:** Summary of Food and Drug Administration (FDA)-approved Invasive Stimulation Approaches.

	Patient Factors (FDA Approval)	Battery Location	MRI Conditional?	Common Adverse Effects [41,58,75]	>50% Reduction in Seizures at 1 year in RCTs (Adult Patients) [26,41,58]	Seizure Free [41,42,62,121]
**VNS**	≥4 years	Chest wall	Yes	Hoarseness (55%) Cough (15%) Pain (15%) Dyspnea 38%)	34%–37%	5% (at 4 months after implant)
**RNS**	≥18 years Refractory to ≥2 ASMs ≥3 seizures per month for ≥3 months	Cranium	No	ICH (4.7%) Lead damage (2.6%) Infection (4%)	44%	16% (for ≥12 months)
**DBS of ANT**	≥18 years ≥6 seizures per month Refractory to ≥2 ASMs and taking 1–4 ASMs	Chest wall	Yes	Implant site pain (23%) Infection (13%) Lead misplacement (8.2%)	43%	16% (for ≥6 months)
